# Efficacy of Pectoral Nerve Block Type II for Breast-Conserving Surgery and Sentinel Lymph Node Biopsy: A Prospective Randomized Controlled Study

**DOI:** 10.1155/2018/4315931

**Published:** 2018-05-15

**Authors:** Doo-Hwan Kim, Sooyoung Kim, Chan Sik Kim, Sukyung Lee, In-Gyu Lee, Hee Jeong Kim, Jong-Hyuk Lee, Sung-Moon Jeong, Kyu Taek Choi

**Affiliations:** ^1^Department of Anesthesiology and Pain Medicine, Asan Medical Center, University of Ulsan College of Medicine, Seoul, Republic of Korea; ^2^Division of Breast and Endocrine Surgery, Department of Surgery, Asan Medical Center, University of Ulsan College of Medicine, Seoul, Republic of Korea

## Abstract

**Objectives:**

The pectoral nerve block type II (PECS II block) is widely used for postoperative analgesia after breast surgery. This study evaluated the analgesic efficacy of PECS II block in patients undergoing breast-conserving surgery (BCS) and sentinel lymph node biopsy (SNB).

**Methods:**

Patients were randomized to the control group (*n*=40) and the PECS II group (*n*=40). An ultrasound-guided PECS II block was performed after induction of anesthesia. The primary outcome measure was opioid consumption, and the secondary outcome was pain at the breast and axillary measured using the Numerical Rating Scale (NRS) 24 hours after surgery. Opioid requirement was assessed according to tumor location.

**Results:**

Opioid requirement was lower in the PECS II than in the control group (43.8 ± 28.5 *µ*g versus 77.0 ± 41.9 *µ*g, *p* < 0.001). However, the frequency of rescue analgesics did not differ between these groups. Opioid consumption in the PECS II group was significantly lower in patients with tumors in the outer area than that in patients with tumors in the inner area (32.5 ± 23.0 *µ*g versus 58.0 ± 29.3 *µ*g, *p*=0.007). The axillary NRS was consistently lower through 24 hr in the PECS II group.

**Conclusion:**

Although the PECS II block seemed to reduce pain intensity and opioid requirements for 24 h after BCS and SNB, these reductions may not be clinically significant. This trial is registered with Clinical Research Information Service KCT0002509.

## 1. Introduction

Breast-conserving surgery (BCS) and sentinel lymph node biopsy (SNB) are surgical methods designed to minimize intraoperative tissue injury, removing the cancer while leaving intact as much of the breast as possible. Moreover, because long-term survival rates are similar in patients undergoing BCS and radical mastectomy [1], the combination of BCS and SNB has become the standard treatment for patients with early-stage breast cancer [2].

Although BCS is minimally invasive surgery, it can lead to significant postoperative pain [3]. Because acute postoperative pain and BCS may be risk factors for persistent pain after breast cancer surgery, it is important to manage postoperative pain in patients undergoing BCS and SNB [4]. A thoracic epidural block used to be regarded as the gold-standard method for managing postoperative pain after breast surgery [5]. However, this technique is associated with serious complications, including intrathecal spread, nerve damage, epidural hematoma, and inadvertent intravascular injection [6]. A recently introduced pectoral nerve block type II (PECS II block) has been found to provide great pain relief and safety in patients undergoing radical mastectomy [7, 8]. Therefore, we hypothesized that the PECS II block may effectively alleviate acute postoperative pain in patients undergoing BCS and SNB. The present study evaluated the analgesic efficacy of PECS II block in patients undergoing BCS and SNB. In addition, this study assessed the efficacy of PECS II block according to breast cancer location and its comparative effects on breast and axillary pain.

## 2. Methods

### 2.1. Patients

This study enrolled patients with early breast cancer scheduled to undergo BCS and SNB between July 2016 and May 2017. The trial was approved by the Institutional Review Board (2016-0738) of Asan Medical Center and was registered at the Clinical Research Information Service (KCT 0002509). All patients provided written informed consent.

Patients were included if they were aged 20–70 years and had American Society of Anesthesiologists (ASA) physical status I and II. Patients were excluded if they had used an anticoagulant, did not cooperate with the study protocol, were allergic to local anesthetics, had serious neurological or psychiatric disorders, or were pregnant or breastfeeding. Patients with one and three incision sites were also excluded. Patients were randomized to two groups according to a computer-generated randomization schedule. Patients in the PECS II group received a PECS II block following the induction of general anesthesia, whereas patients in the control group did not receive any regional analgesia during the perioperative period.

### 2.2. Process of Anesthesia and Analgesia

Anesthesia was induced by administration of propofol (2 mg/kg). After the patient lost consciousness, rocuronium (0.6 mg/kg) was injected for smooth tracheal intubation. Desflurane and remifentanil were also used for induction. Remifentanil was administrated via target-controlled infusion using Orchestra (Fresenius Vial, Brezins, France). Anesthesia was maintained with desflurane 5-6% in 50% oxygen and 2–2.5 ng/ml of effect-site remifentanil concentration. After surgery, the patients were moved to the postanesthetic care unit (PACU) and administered fentanyl (0.4 *µ*g/kg) when in need of analgesics or when analgesia was insufficient (Numerical Rating Scale (NRS) ≥ 4). Injection of fentanyl in the PACU was repeated until the patient was satisfied with analgesia. Upon being moved to the general ward, patients were administered 30 mg of the nonsteroidal anti-inflammatory drug (NSAID) ketorolac to reduce postoperative pain. Patients with sustained inadequate analgesia were administered meperidine 25 mg or tramadol 50 mg until 24 hours after surgery.

### 2.3. Ultrasound-Guided PECS II Block

**Figure 1:**
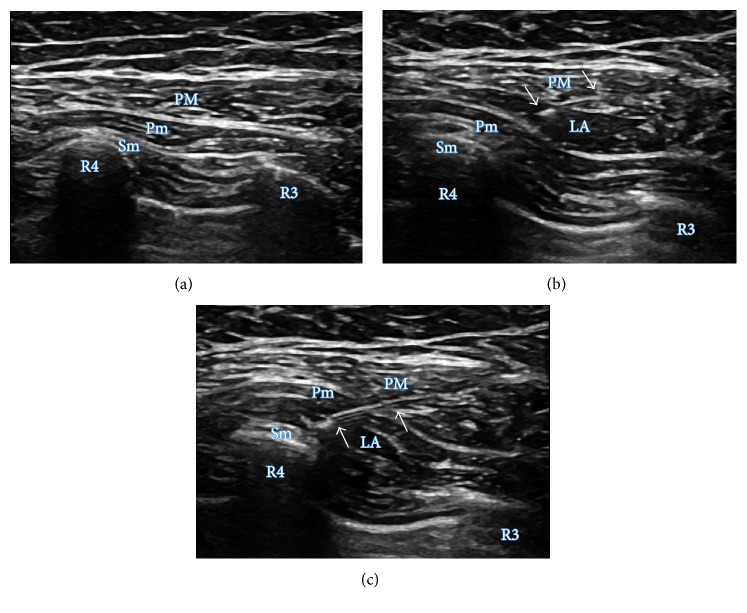
Ultrasound images of the introduction of a PECS II block. (a) Target areas of the PECS II block. (b) First injection of the PECS II block, showing spreading of local anesthetic in the interfascial plane between the pectoralis major and pectoralis minor muscles. (c) Second injection of the PECS II block, showing spreading of local anesthetic in the interfascial plane between the pectoralis minor and serratus anterior muscles. PM, pectoralis major muscle; Pm, pectoralis minor muscle; SA, serratus anterior muscle; LA, local anesthetic; R3, third rib; R4, fourth rib. The arrow indicates the 23-gauge Quincke needle.

Ultrasound-guided PECS II block was performed following general anesthesia to obviate any pain and anxiety associated with a regional block in conscious patients. This procedure was conducted according to the techniques described by Blanco et al. and therefore also included a PECS I block [9]. Patients were placed in the supine position on an operating table with their arm abducted. After sterile preparation for the procedure, a 12 MHz linear ultrasound probe (NextGen LOGIQ e Ultrasound, GE Healthcare, USA) was positioned below the lateral third of the clavicle. The positions of the axillary artery and vein were confirmed, and the ultrasound probe was moved inferolaterally until the pectoralis major and minor and the serratus anterior muscles were identified in one plane at the level between the third and fourth ribs. A 23-gauge Quincke type spinal needle (TaeChang Industrial Co., Korea) was advanced in plane view of the ultrasound probe from the medial to lateral direction until it reached the interfascial plane between the pectoralis major and minor muscles. After the position of the needle tip was confirmed, 10 ml of 0.25% ropivacaine was administered. The needle was subsequently advanced further until its tip was located in the interfascial plane between the pectoralis minor and serratus anterior muscles, and an additional 20 ml of 0.25% ropivacaine was administered above the serratus anterior muscles (Figure 1). All of these nerve block procedures were performed by two anesthesiologists who were proficient and experienced in ultrasound-guided PECS II block.

### 2.4. Outcome Measures and Data Collection

**Figure 2:**
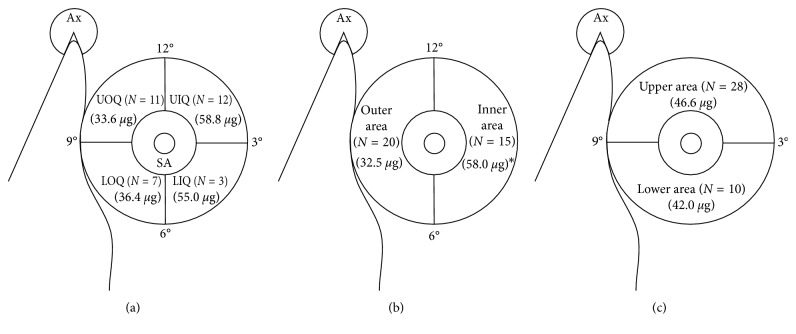
Opioid consumption as a function of breast cancer location. (a) Opioid consumption according to the quadrants of the breast. Patients with cancers located at 12, 3, 6, and 9 o'clock were not excluded because of the ambiguity of location. UOQ, upper outer quadrant; UIQ, upper inner quadrant; LOQ, lower outer quadrant; LIQ, lower inner quadrant; SA, subareolar; Ax, axilla; *N*, number of patients; values within parentheses denote mean fentanyl consumption. (b) Opioid consumption according to tumor location in the outer and inner areas of the breast, as determined by a line connecting the 12 o'clock and 6 o'clock positions. Patients with cancers located at 12 o'clock and 6 o'clock were not excluded, ^∗^*p* value < 0.05. (c) Opioid consumption according to tumor location in the upper and lower areas of the breast, as determined by a line connecting the 3 o'clock and 9 o'clock positions. Patients with cancers located at 3 o'clock and 9 o'clock were not excluded.

All baseline and postoperative measurements were evaluated by an independent physician who was blinded to treatment allocation. Postoperative pain intensity was assessed using a single 11-point NRS (in which 0 = no pain and 10 = worst pain imaginable). The NRS was measured separately on the breast and axilla. To obtain a valid NRS value after the operation, all participants were instructed before the procedure about how to check the NRS correctly. Doses of all opioids administered to patients were converted to intravenous fentanyl equianalgesic doses according to published conversion factors (intravenous fentanyl 100 *μ*g = meperidine 100 mg = tramadol 100 mg) [10]. Analgesic consumption and the NRS were measured 0, 0.5, 1, 2, 6, 9, 18, and 24 hours after the end of surgery. Opioid requirements were analyzed as a function of breast cancer location (quadrants, outer and inner areas, and upper and lower area; Figure 2). Complications associated with the PECS II block and with analgesics, such as pneumothorax, hematoma, nausea, vomiting, and urinary retention, were recorded. Vital signs (e.g., oxygen saturation, blood pressure, heart rate, and electrocardiography) were measured during the first 24 hours postoperatively. Differences in mean blood pressure and heart rate from before to after the incision were calculated. The sensory level of the block was evaluated using the cold test, performed by an independent physician after the operation.

A medical bandage was applied to the site of needle insertion in the PECS II group after the operation. To ensure patients were unaware whether the PECS II block had been performed, a bandage was also applied to a similar site in the control group.

The primary study outcome was the difference in 24-hour postoperative opioid consumption between the PECS II and control groups. Secondary outcomes included the NRS for each breast and axilla, changes in vital signs at incision, opioid requirements according to breast cancer location, side effects of analgesics (nausea, vomiting, dizziness, pruritus, sleeping tendency, urinary retention, and respiratory depression), and complications of the PECS II block.

### 2.5. Statistical Analysis

The sample size was calculated based on our pilot study. If the mean ± standard deviation (SD) difference in opioid consumption between the PECS II and control groups was 48 ± 64 *μ*g of fentanyl, with a significance level of 0.05 and a power of 0.9, and assuming a dropout rate of 5%, then 80 patients (40 per group) should be sufficient. Data were analyzed using the Statistical Package for the Social Sciences (SPSS version 21.0, SPSS Inc., Chicago, IL). Normal distribution of data was tested using the Kolmogorov–Smirnov test. Normally distributed continuous data were reported as mean ± SD and compared using Student's t-tests. Nonparametric continuous data were presented as median and interquartile range and compared using Mann–Whitney *U* tests. Categorical data were presented as numbers and percentages and compared using the chi-square test or Fisher's exact test. Opioid consumption as a function of breast cancer location was determined using the Kruskal–Wallis test. A *p* value below 0.05 was considered statistically significant.

## 3. Results

**Figure 3:**
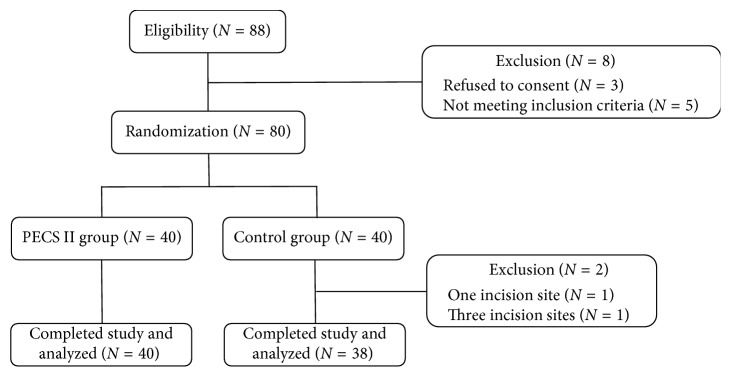
Study flow chart.

Eighty patients were enrolled in this study, 40 in the PECS II group and 40 in the control group. Two patients in the control group, one with a single incision site and one with three incision sites, were excluded (Figure 3). The baseline demographic and clinical characteristics of the two groups are shown in Table 1. As expected, the changes in mean blood pressure and heart rate (from before to after the incision) were greater in the control than in the PECS II group. The side effect rates of analgesics were similar in the two groups (Table 2).

Opioid consumption during the first 24 hours after surgery was significantly lower in the PECS II group than in the control group (43.8 ± 28.5 *µ*g versus 77.0 ± 41.9 *µ*g, *p* < 0.001), but the frequency of rescue NSAIDs did not differ between these groups. The rates of side effects of analgesics were also similar in the two groups (Table 2). Analysis of patients in the PECS II group showed that opioid consumption was unrelated to the quadrant in which the breast cancer was located, that is, whether the tumor was located in the upper or lower area of the breast. However, opioid consumption was significantly greater in PECS II patients with tumors in the inner area than in the outer area of the breast (58.0 ± 29.3 *µ*g versus 32.5 ± 23.0 *µ*g, *p*=0.007; Figure 2).

**Figure 4:**
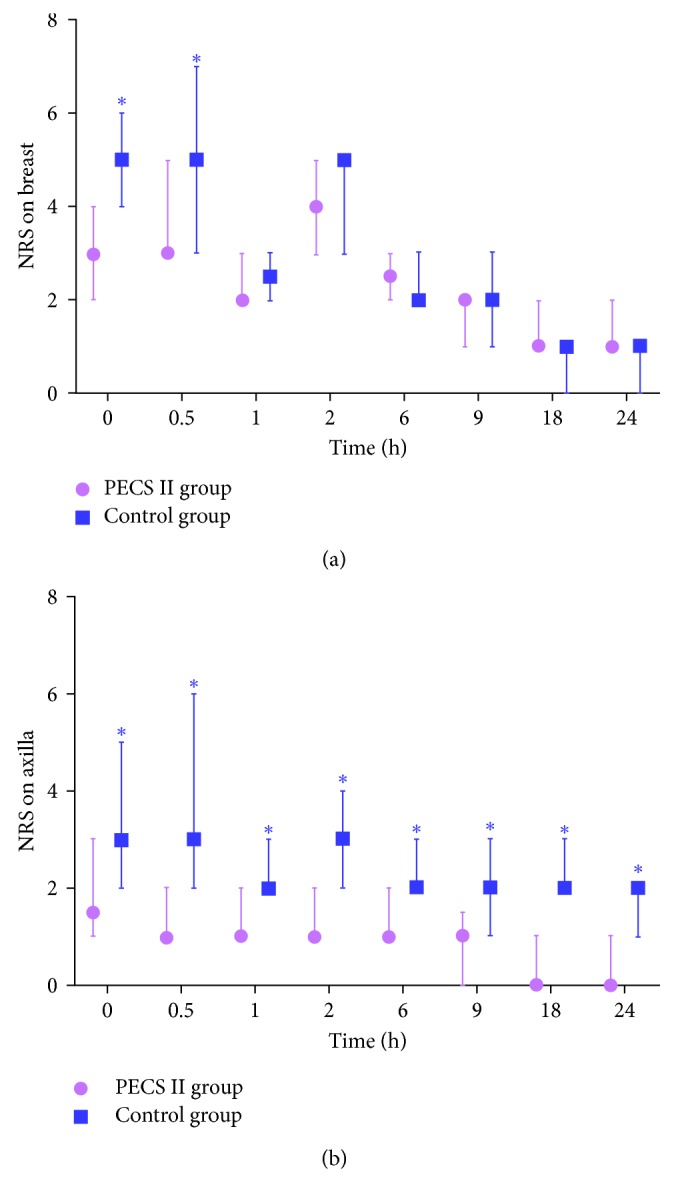
NRS of the breast (a) and axilla (b) in the PECS II and control groups. Data are expressed as the median (interquartile range). ^∗^*p* value < 0.05.

Mean NRS value of the breast was significantly lower in the PECS II than in the control group at 0 (3.0 ± 1.5 versus 4.9 ± 1.6, *p* < 0.001) and 0.5 (3.6 ± 1.5 versus 5.1 ± 1.8, *p* < 0.001) hours after the procedure. Median NRS value of the breast was not statistically lower in the PECS II than in the control group starting 1 hour after surgery. Median NRS value of the axilla, however, was significantly lower in the PECS II than in the control group throughout the first 24 hours after surgery (Figure 4). None of these patients reported complications associated with the PECS II block.

## 4. Discussion

This study had two main findings. First, although the PECS II block seemed to reduce pain severity and opioid consumption in patients undergoing BCS and SNB, it may not have clear clinical efficacy. Second, the PECS II block had a significantly greater effect in reducing axillary pain.

Since the introduction of PECS II block, several randomized controlled trials have shown that the PECS II block is effective in reducing pain in patients undergoing mastectomy [7–9, 11, 12]. To our knowledge, the present study is the first to test the efficacy of PECS II block only in patients undergoing BCS and SNB. The mean difference in opioid requirement between the two groups was only 33.2 *μ*g of fentanyl. In other studies of interfascial plane block, the minimum difference in opioid consumption between the nerve block and control groups was 13 mg of morphine or 100 *μ*g of fentanyl [13, 14]. The 33 *μ*g difference in fentanyl consumption over 24 hours in the present study was less than 2 *μ*g per hour, a quantitative difference lower than in other studies of regional analgesia. Similar to our results, two previous studies also found that the mean differences in 24-hour postoperative morphine consumption between the PECS II and control groups were 5.81 mg and 3.67 mg [12, 15]. Moreover, the frequency of rescue NSAIDs and the side effects of analgesics in the present study did not differ in the PECS II and control groups. These findings indicate that, although the PECS II block seemed to statistically significantly reduce rescue analgesic use, the difference may not have clinical significance. The present study also showed that the breast pain score was lower in the PECS II group than in the control group only for the first 30 min postoperatively. Moreover, the median difference in the NRS score between these groups was less than 1 at all other time points. This difference did not meet the threshold for a minimal clinically important difference in acute postoperative pain (i.e., a difference ≥10 on the 100 mm pain visual analogue scale) [16]. Therefore, the PECS II block appeared not to be clinically useful.

**Figure 5:**
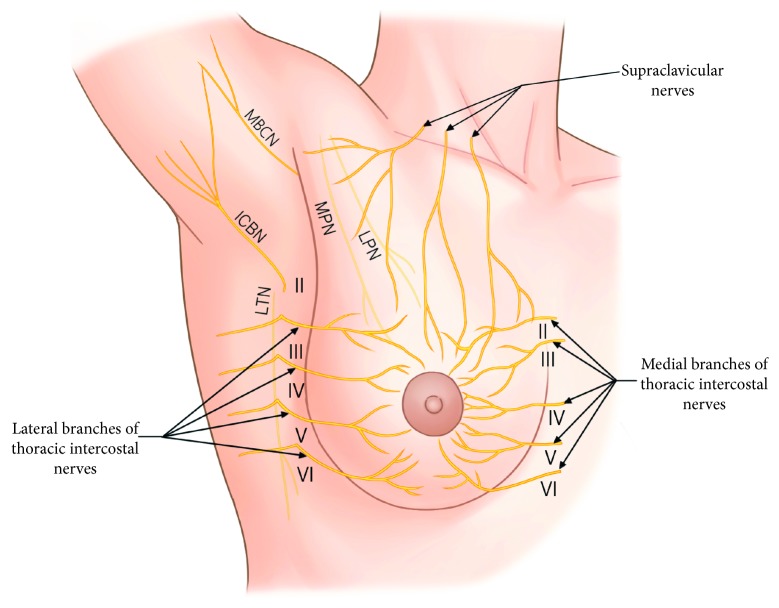
Diagrammatic representation of the nerves innervating the female breast and axilla. MPN, medial pectoral nerve; LPN, lateral pectoral nerve; MBCN, medial brachial cutaneous nerve; ICBN, intercostobrachial nerve; LTN, long thoracic nerve.

**Figure 6:**
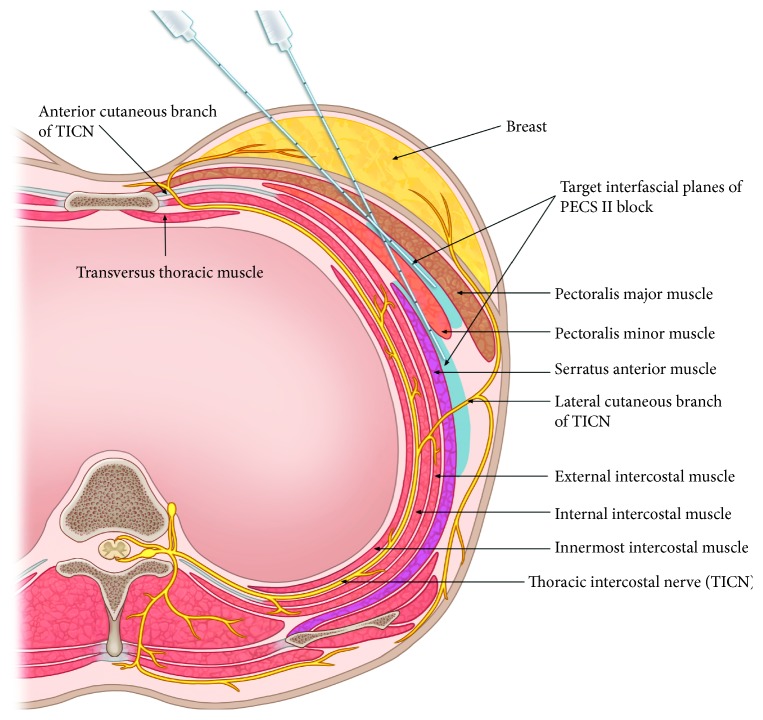
Illustration of target areas of the PECS II block. This agent can block the lateral cutaneous branches of the TICN in the interfascial plane between the pectoralis minor and serratus anterior muscles but cannot block the anterior cutaneous branch of the TICN.

The lack of clinical significance of the PECS II block may have been due to its inability to block all the nerves innervating the breast. The breast is innervated by multiple nerve branches, including the lateral and anterior cutaneous branches of the second to sixth thoracic intercostal nerves (TICNs) and several branches of the supraclavicular nerves (Figure 5) [17, 18]. Thus, it is doubtful whether a single blocking method can provide adequate analgesia throughout the entire breast area. The targets of PECS II block include the medial and lateral pectoral nerves, including the lateral cutaneous branches of the TICNs (Figure 6). Local anesthetics cannot reach the anterior cutaneous branches of the TICNs by piercing the external and internal intercostal muscles. Therefore, they cannot block anterior cutaneous branches of the second to sixth TICNs and branches of the supraclavicular nerves. Although several recent studies have also mentioned these limitations of the PECS II block [15, 19, 20], those studies, in contrast to ours, did not demonstrate these limitations.

Additionally, we evaluated opioid requirements associated with tumor location in the breast (quadrant, outer/inner, and upper/lower areas). Opioid consumption did not differ significantly by breast tumor quadrant or in patients with tumors in the upper and lower areas. However, opioid requirements were greater in patients with tumors in the inner area than in the outer area of the breast. The inner area is primarily innervated by the anterior cutaneous branches of the TICN, whereas the outer area is primarily innervated by the lateral cutaneous branches of the TICN. Therefore, our finding suggests that the PECS II block could block the lateral, but not the anterior, cutaneous branches of the TICN.

Interestingly, axillary pain scores were significantly lower in the PECS II group than in the control group for up to 24 hours after surgery. The median difference in NRS between these groups was >1.5 at most evaluation times. These findings indicated that the PECS II block could be useful as regional analgesia for patients undergoing SNB. Local anesthetic administered into the interfascial plane likely reached the axilla via an axillary port, easily blocking the intercostobrachial and medial brachial cutaneous nerves, which innervate the axillary area. The spread of local anesthetic into the axilla has been demonstrated by dissection of cadavers and contrast distribution [9, 21]. The pectoral nerve block was also found to be beneficial for axillary surgery [22]. Consequently, the PECS II block may be effective at alleviating axillary pain.

In agreement with previous studies, no complications were associated with the PECS II block procedure. A PECS II block is conducted while patients are in the supine position, and the needle is manipulated relatively easily. Moreover, the target areas of a PECS II block are distant from the pleura and epidural space, but relatively close to the skin surface (Figure 6). Although the thoracoacromial artery may be present at the interfascial plane, it is easily visualized by ultrasonography. Direct intravascular injection of local anesthetics is performed very rarely due to a lack of vasculature at the interfascial plane [23, 24]. Therefore, a PECS II block seems to be a safe procedure.

This study had several limitations. First, the PECS II block was performed following the induction of general anesthesia to reduce procedural pain and anxiety, which may have affected postoperative pain [25]. Sensory level tests were performed in the PACU after the operation, with all patients in the PECS II group showing positive reactions on the cold test. However, in contrast to findings in a previous study, our patients did not express exact dermatome against cold tests [20], suggesting that wound dressing and a surgical brassiere may have interfered with these sensory examinations. Other reasons for inaccurate responses to sensory level tests include postoperative pain, the sedative effect of opioids, and anesthetic hangover. However, we speculated that the PECS II block was successfully performed based on the changes in mean blood pressure and heart rate during the incision and the positive reactions in the cold test. Consequently, this study did not present sensory test data. A second limitation of this study was our inability to perform a double-blind, placebo-controlled study. However, the patients and investigators were blinded to group assignment, suggesting that the lack of ability to perform a placebo-controlled study had little influence on the study outcomes.

In conclusion, although the PECS II block reduced pain intensity and opioid requirements for 24 hours in patients who underwent BCS and SNB, PECS II block may not be clinically useful. Because PECS II block could not completely block all the nerves innervating the breast, including the anterior cutaneous branch of the TICN, it could not provide complete postoperative analgesia after BCS and SNB. The PECS II block seemed to be more efficient at reducing axillary pain than breast pain. Therefore, PECS II block may lack the ability to provide sufficient postoperative analgesia after breast surgery.

## Figures and Tables

**Table 1:** Baseline demographic and clinical characteristics of study subjects.

	PECS II group (*n*=40)	Control group (*n*=38)
Age (years)	45.4 ± 9.9	45.2 ± 11.9
BMI (kg/m^2^)	22.8 ± 2.8	23.9 ± 3.1
ASA class (I/II)	36 (90.0%)/4 (10.0%)	29 (76.3%)/9 (23.7%)
Neoadjuvant CTx	6 (15.0%)	6 (15.8%)
Surgical time (min)	93.5 ± 19.9	89.7 ± 24.9
Intraoperative remifentanil dosage (*μ*g)	491.0 (440.0; 571.0)	477.0 (420.0; 600.0)
Tumor location (left/right)	14 (35.0%)/26 (65.0%)	21 (55.3%)/17 (44.7%)
Tumor location (quadrant)		
UOQ/LOQ	11 (27.5%)/7 (17.5%)	15 (39.5%)/7 (18.4%)
UIQ/LIQ	12 (30.0%)/3 (7.5%)	7 (18.4%)/3 (7.9%)
12 o'clock/6 o'clock	5 (12.5%)/0 (0.0%)	1 (2.6%)/1 (2.6%)
3 o'clock/9 o'clock	0 (0.0%)/2 (5.0%)	2 (5.2%)/1 (2.6%)
Subareolar	0 (0.0%)	1 (2.6%)

Data are expressed as mean ± SD (standard deviation), number (%), or median (interquartile range). BMI, body mass index; ASA, American Society of Anesthesiologists Physical Status Classification; CTx, chemotherapy; UOQ, upper outer quadrant; LOQ, lower outer quadrant; UIQ, upper inner quadrant; LIQ, lower inner quadrant.

**Table 2:** Opioid requirements, frequency of rescue NSAIDs, and incidence of side effects of analgesics in the PECS II and control groups during the 24 hours after the operation.

	PECS II group (*n*=40)	Control group (*n*=38)	*p* value
Total opioid requirements (*μ*g)	43.8 ± 28.5	77.0 ± 41.9	<0.001
Frequency of rescue NSAIDs	1.0 (0.0; 1.0)	1.0 (1.0; 1.0)	0.213
MBP after incision − MPB before incision (mmHg)	5.0 (1.0; 10.5)	16.0 (9.0; 24.0)	<0.001
HR after incision − HR before incision (beats per minute)	0.0 (−2.0; 2.5)	3.0 (1.0; 5.0)	0.002
Side effects of analgesics (%)	7 (17.5%)	10 (26.3%)	0.504

Data are expressed as mean ± SD (standard deviation), median (interquartile range), or number (%). NRS, Numerical Rating Scale; NSAID, nonsteroidal anti-inflammatory drug; MBP, mean blood pressure; HR, heart rate.

## Data Availability

The authors will provide data upon request to the first author (Doo-Hwan Kim, e-mail: knaaddict@gmail.com).
